# Association between obesity and early tooth eruption in adolescents: Findings from a popu-lation-based cohort study in southern Brazil 

**DOI:** 10.4317/jced.60340

**Published:** 2023-10-01

**Authors:** Nicássia-Cioquetta Lock, Ângela-Dalla Nora, Nailê Damé-Teixeira, Carolina-Doege Brusius, Marisa Maltz, Luana-Severo Alves

**Affiliations:** 1 Federal University of Santa Maria - UFSM, School of Dentistry, Department of Restorative Dentistry, Santa Maria, RS, Brazil; 2 Department of Stomatology, School of Dentistry, Federal University of Santa Maria, Santa Maria, RS, Brazil; 3 Department of Dentistry, University of Brasilia, Brasilia, Brazil; 4 Federal University of Rio Grande do Sul, Faculty of Odontology, Department of Social and Preventive Dentistry, Porto Alegre, RS, Brazil

## Abstract

**Background:**

Obesity is a prevalent chronic condition affecting children and adults worldwide, and it seems to influence the timing of tooth eruption. The aim of this study was to assess the as-sociation between weight status at age 12 and the eruption of permanent teeth at ages 12 and 14-15 among schoolchildren from southern Brazil.

**Material and Methods:**

A cross-sectional survey was conducted in Porto Alegre, southern Brazil, and included a representative sample of 1,528 12-year-old schoolchildren. After 2.5 years, 801 individuals were reexamined. Baseline data collection included a questionnaire, the record-ing of anthropometric measures (height and weight), and clinical examination to register the number of erupted permanent teeth. At follow-up, the eruption stage of second perma-nent molars was recorded. Statistical analysis used Poisson regression.

**Results:**

Overweight and obese individuals were 32% and 88% more likely to have com-plete permanent dentition at age 12, respectively (overweight, PR=1.32, 95%CI=1.13-1.55; obese, PR=1.88, 95%CI=1.75-2.02). Obese 12-year-olds were more likely to present erupt-ed #17, #27, #37, and #47 at age 12 and to present completely erupted second molars at age 14-15 than normal weight ones.

**Conclusions:**

This population-based study found a significant association between over-weight/obesity at age 12 and early tooth eruption at ages 12 and 14-15 among schoolchil-dren from southern Brazil.

** Key words:**Tooth eruption, Obesity, Permanent teeth, Epidemiology.

## Introduction

The eruption of permanent teeth is a continuous, orderly, sequential, and age-specific biological process, in which the teeth emerge through the jaws and overlap the mucosa to enter the oral cavity (1,2). Some factors can exert influence on eruption patterns including sex, genetics, ethnicity, geographical location, hormonal factors, socioeconomic status, nutrition, and growth (3).

In addition to these factors, previous literature has suggested that obesity may be associated with early tooth eruption. The systematic review by Mohamedhussein *et al*. (4) evaluated the body of evidence on the relationship between obesity and eruption of permanent molars in children under 16 years of age as compared to normal-weight children (4). After assessing six cross-sectional studies (5-9) and one longitudinal survey,11 the authors concluded that a positive correlation between overweight/obesity and earlier tooth emergence can be noted from the existing literature; however, main risk of bias arises from the cross-sectional nature of included studies and lack of control of potential confounders. The only longitudinal study assessing this relationship available in the literature was conducted by Sánchez-Pérez *et al*. in a convenience sample of 88 Mexican children (10). Therefore, further high-quality evidence is required to elucidate this association, mainly derived from longitudinal studies.

After the cited systematic review, other studies were published with conflicting results. While some authors found that increased body weight was associated with early tooth eruption and advanced dental age (11-14), others revealed contradictory evidence, such as lack of association (15) or an inverted relationship (16). Paz-Cortez, 2022 when evaluating a sample of patients between 4-14 years old, showed that body mass index (BMI) did not influence tooth eruption (15). In the same way, tooth eruption was delayed with increasing BMI in Indian children aged 6-7 years (16).

Considering the need for further evidence and the scarcity of longitudinal studies on this topic, the aim of this study was to assess the association between weight status at age 12 and the eruption of permanent teeth at ages 12 and 14-15 among adolescents from southern Brazil. This knowledge is clinically relevant considering the prevention of dental caries and malocclusions at target ages.

## Material and Methods

-Study design and sample

Initially, a representative sample of the population of 12-year-old schoolchildren from Porto Alegre, southern Brazil was selected using a multistage probability sampling strategy. The primary sampling unit consisted of five geographical areas organized according to the municipal water fluoridation system. Within each area, the schools were randomly selected proportional to the number of private and public schools (42 schools: 33 public and 9 private).

**Figure 1:**
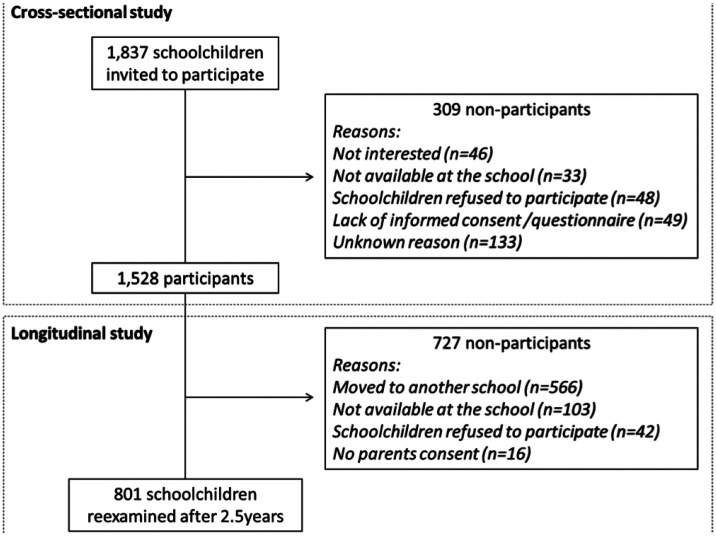
Study flowchart.

Schoolchildren born in 1997 or 1998 were then randomly selected proportional to the school size. The parameters used for the sample calculation were caries prevalence of 60%,12 (17) with a precision level of ±3% for the 95% confidence interval, and assuming a design effect of 1.3. The minimum sample size required for this study was 1,331 school-children. A nonresponse error of 40% was added, and a final sample size of 1,837 was es-timated. The original sample included in the cross-sectional study was composed of 1,528 12-year-old schoolchildren. After a mean period of 2.5 years (SD=0.3), it was possible to reexamine 801 schoolchildren (14.8±0.5 years old), representing 52.4% of the sample initially examined, as show in Figure 1. Baseline characteristics of followed schoolchildren were compared with those lost to follow-up and no significant difference was observed regarding sex, skin color, socioeconomic status, weight status, type of permanent dentition (*p*>0.05, chi-square test) and number of permanent teeth (*p*<0.05, Wald test).

-Data collection 

Baseline data collection was carried out from September 2009 to December 2010. A structured questionnaire containing questions on socio-demographic information was sent to the parents/legal guardians of the selected students.

Anthropometric measures (weight and height) were collected by a single researcher (NDT) and used to assess schoolchildren’s weight status. Weight was measured using a 150 kg digital scale, and two readings were made. A third assessment was taken if a difference >0.3 kg was observed between measurements. The mean of the two closest measurements was used to calculate BMI. Height was measured to the nearest full centimeter using an inelastic metric tape attached to a flat wall with no footer. The students were wearing light clothing and no shoes.

Clinical examination was conducted at the schools. With the students in a supine po-sition and under artificial light, a single examiner (NDT) recorded the number of erupted permanent teeth. A tooth was defined as having erupted when any part of the crown has perforated the oral mucosa and was clinically visible. No radiographic examination was performed. 

Follow-up clinical examinations were performed between August 2012 and May 2013 by another examiner (CDB). Schoolchildren were reexamined at the schools, follow-ing the same protocol previously described. The eruption stage of second permanent molars was recorded as follows: 1) occlusal surface partially erupted; 2) occlusal surface fully erupted, but more than half of the tooth buccal surface was covered with gingival tissue; 3) the occlusal surface fully erupted, and less than half of the tooth buccal surface was covered with gingival tissue; and 4) full occlusion (18).

-Data analysis

The primary outcome of this study was type of dentition, defined as mixed (children presenting at least one deciduous tooth), incomplete permanent (children presenting only per-manent teeth, up to 27 erupted teeth), or complete permanent (children presenting 28 erupted permanent teeth). For the risk assessment analysis, type of dentition was converted into a binary variable defining the presence of complete permanent dentition (mixed/incomplete permanent versus complete permanent). Secondary outcomes were the number of erupted permanent teeth, the presence of erupted second molars at age 12, and the complete eruption of second molars at age 14-15.

BMI-for-age Z-scores were calculated using specific software (AnthroPlus, WHO, Geneva, Switzerland). BMI-for-age Z-score is a measure of the standard deviation (SD) away from standardized mean BMI. It is considered one of the most appropriate measures of weight in children and adolescents because it accounts for the wide, natural variation in growth. Using cutoffs recommended by the WHO, (19) the sample was categorized as fol-lows: normal weight (BMI-for-age Z-score ≤ +1 SD), overweight (BMI-for-age Z-score > +1 SD to ≤ +2 SD), or obese (BMI-for-age Z-score > +2 SD).

Skin color was dichotomized as white and non-white. Socioeconomic status was as-sessed according to the standard Brazilian economic classification, and families were classi-fied as low (≤ 13 points), mid-low (≥ 14 to≤ 22 points), mid-high (≥ 23 to≤ 28 points) or high (≥ 29 points) socioeconomic status.

Data analysis was performed using survey commands that took into account the survey design, including clustering, weighting, and robust variance estimation. Given this discrepancy between the study participants and non-respondents, a weighted variable based on information provided by the Primary Education School Census of 2010 was used in the statistical analysis to minimize non-response bias. Statistical analysis was performed using STATA software (Stata 14.2 for Windows; Stata Corporation, College Station, TX, USA) and the level of significance was set at 5%.

Preliminary analysis comparing the type of dentition and the number of erupted permanent teeth by categories of sex, skin color, socioeconomic status, and weight status was performed using the chi-square test and the Wald test, respectively.

The association between predictor variables and the presence of complete perma-nent dentition was assessed using Poisson regression models. Unadjusted and adjusted prev-alence ratios (PR) and their 95% confidence intervals (CI) were estimated. Weight status was considered the main predictor variable. Sex, skin color, and socioeconomic status were included in the adjusted model as controlling variables.

Poisson regression models were also used to assess the association between weight status at age 12 and (i) the presence of erupted second permanent molars at age 12 and (ii) the complete eruption of second permanent molars at age 14-15. In both analyses, unad-justed and adjusted analyses were performed, as previously described.

-Ethical aspects

The study protocol was approved by the Federal University of Rio Grande do Sul Research Ethics Committee (299/08) and by the Municipal Health Department of Porto Alegre Re-search Ethics Committee (process n° 001.049155.08.3/register n° 288 and process nº 001.028618.12.2/register nº 807). All participants and their parents/legal guardians provid-ed written informed consent.

## Results

Table 1 shows the sample distribution, type of dentition, and the number of erupted permanent teeth at the age 12 by predictor variables. Among the 1,528 schoolchildren, 952 (62.3%) had permanent dentition whereas 576 (37.7%) had mixed dentition. This school-children population had an average of 24.5 (95%CI=23.7-25.3) erupted permanent teeth, ranging from 11 to 28. It was observed a significant gradient among weight categories, with increasing proportion of individuals with permanent dentition and increasing number of erupted permanent teeth with increasing weight status. Significant differences were also found for sex and skin color, with girls and non-white schoolchildren showing permanent dentition more often than boys and white ones. Girls had also a significant higher number of erupted permanent teeth. No relationship was found with socioeconomic status.

**Table 1:**
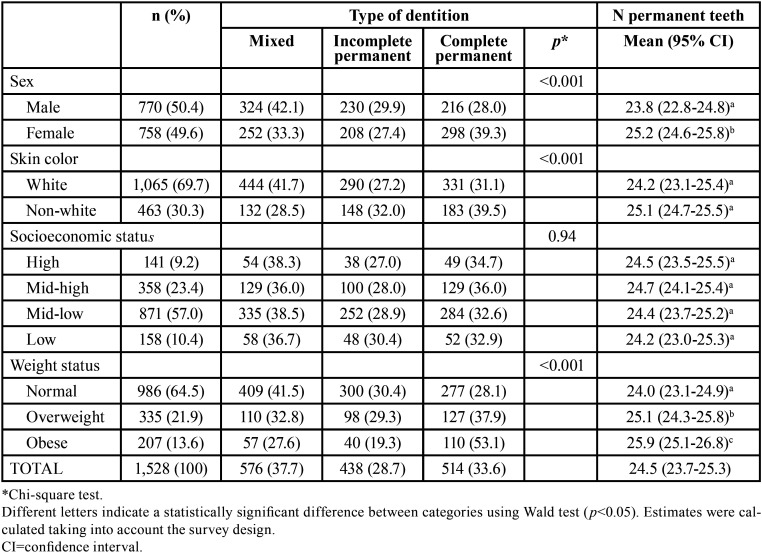
Sample distribution, type of dentition, and the number of erupted permanent teeth at age 12 by predictor variables (n=1,528 – baseline assessment).

Table 2 shows the association between predictor variables and the presence of complete permanent dentition at age 12. Overweight and obese individuals were 32% and 88% more likely to have complete permanent dentition at age 12 than normal weight schoolchil-dren, respectively (overweight, adjusted PR=1.32, 95% CI=1.13-1.55; obese, adjusted PR=1.88, 95% CI=1.75-2.02). The inclusion of other variables in the adjusted models had a negligible effect on the estimates. In addition, significant associations were found for sex (girls) and skin color (non-white).

**Table 2:**
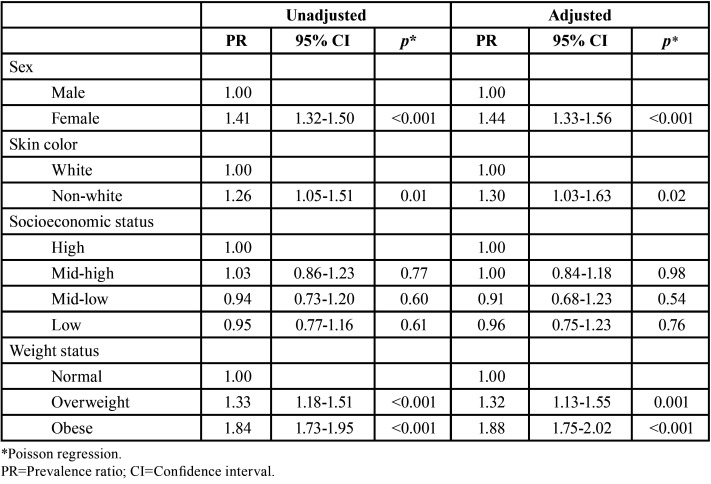
Association between predictor variables and the presence of complete permanent dentition at age 12 (n=1,528 – baseline assessment).

The association between weight status and the presence of erupted second perma-nent molars at age 12 is shown in Table 3. The proportion of erupted second permanent molars increased as the weight status increased (*p*<0.001). In both unadjusted and adjusted analysis, the weight status was significantly associated with the presence of erupted teeth #17, #27, #37 and #47. Overweight and obese schoolchildren were consistently more likely to have erupted second permanent molars. As shown in Table 4, the proportion of school-children with completely erupted second permanent molars at age 14-15 increased as the weight status at age 12 increased (p≤0.05). Obese 12-year-old schoolchildren were more likely to present completely erupted #17, #27, #37 and #47 at age 14-15 than their counterparts with normal weight.

**Table 3:**
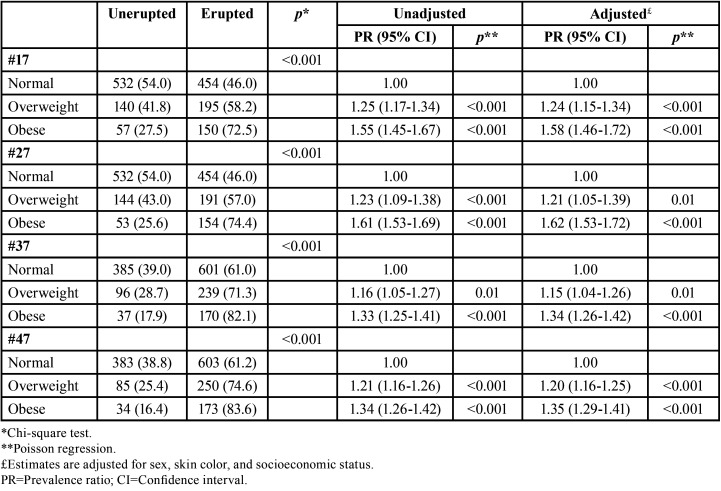
Association between weight status and the presence of erupted second permanent molars at age 12 (n=1,528 – baseline assessment).

**Table 4:**
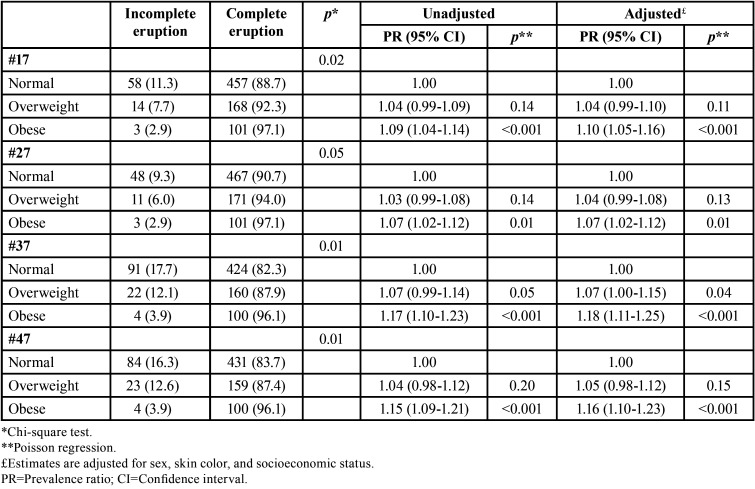
Association between weight status at age 12 and the complete eruption of second permanent molars at age 14-15 (n=801 – follow-up assessment).

## Discussion

This population-based study was conducted to investigate whether the weight status at age 12 could influence the eruption of permanent teeth at ages 12 and 14-15 among school-children from southern Brazil. Our main finding was that overweight and obese school-children consistently had an earlier eruption of permanent dentition than normal weight ones, irrespective of the outcome assessed. To the best of our knowledge, this is the first population-based study addressing this issue.

According to the World Health Organization (WHO), overweight and obesity are defined as a condition of abnormal or excessive fat accumulation in adipose tissue and over 340 million children and adolescents aged 5 to 19 years were overweight or obese in 2016 (20). The biological mechanisms explaining the association between obesity and early tooth eruption is not well understood (4). Obesity is characterized by the presence of inflammato-ry reactions, increased cytokines, exposure to androgens and stimulation of gonadotropin secretion, triggering puberty (21). This way, obesity during childhood may influence pu-bertal onset time and sex hormone levels, (22) mainly among girls. In addition, obesity leads to metabolic changes, such as increased insulin secretion and growth factor, which can also play a role in the early tooth eruption (23). A recently published review brought some light on the orchestra of genes and molecules that possible may help to elucidate the molecular mechanisms linking obesity and tooth eruption (11).

We found a positive association between obesity and early tooth eruption, which is in agreement with studies conducted in Jordan, Hong Kong, Czech Republic, EUA, India, Spain and Brazil (5-7,9). The systematic review by Mohamedhussein *et al*. (4) showed the association between obesity and eruption time of first and second permanent molars in chil-dren under 16 years of age, and indicated that obese 12-year-olds were more likely to have one more erupted tooth than their counterparts. A limitation of this review is that the major-ity of the included studies used a cross-sectional design, thus impeding conclusions on the causative relationship. In the only longitudinal study available in the literature, after as-sessing data from 88 Mexican children that remained in the follow-up, the authors detected a higher eruption rate as the children’s BMI increased over time. In addition, overweight children had about five more permanent teeth than children in the thin group (21.6 versus 15.9, respectively) (10). In the present study, we found that obese schoolchildren were more likely to have complete permanent dentition and erupted second molars at age 12 as well as completely erupted second molars at age 14-15, supporting the previous literature.

Our results are in disagrement with those showed by Anu, 2020 who showed that tooth eruption was delayed with an increase in BMI (16). However, these results are based on a significant correlation between BMI and eruption status of permanent mandibular cen-tral incisors. The same correlation was not found when permanent mandibular molars were considered. In the same way, Paz-Cortez 2022 showed that BMI category did not influence tooth eruption among children betweem 4 and 14 years of age (15).

In this study, sociodemographic factors such as sex and skin color were significantly associated with early eruption. As previously shown by other studies investigating the relationship between obesity and tooth eruption,(5,7,9) we also found that girls had significant-ly more erupted permanent teeth than boys, being 44% more likely to have complete per-manent dentition at age 12 than boys (adjusted PR=1.44, 95% CI=1.33-1.56, *p*<0.001). This is a conceivable finding since girls tend to be more advanced over boys in many aspects of growth and development, such as appearance of calcification centers, epiphyseal union, and appearance of secondary sexual characteristics; and the same occurs with dental maturity, in terms of both calcification (24) and eruption (25). Another finding was that non-white chil-dren were 30% more likely to have complete permanent dentition at age 12 than those of white skin color (adjusted PR=1.30, 95%CI=1.03-1.63, *p*=0.02). Although this relationship has not been well understood, it has been previously found in other studies (7,8,26,27).

When permanent teeth erupt earlier in overweight/obese children, they will be at a high risk for caries development sooner than normal-weight children. In this context, it is important to emphasize that the eruption stage of first and second permanent molars has been recognized as a major risk period for caries development (12,28), which can be attributed to the long eruption stage of these teeth, 28 and to the greater plaque accumulation that occurs in partially erupted molars than in fully erupted molars (12,28). Further-more, overweight/obese children may need orthodontic intervention sooner than normal weight ones, and the timing of assessment for orthodontic treatment needs may require modification in this sort of patients, as discussed by Mohamedhussein *et al*. (4). Therefore, knowledge on the accelerated dental maturation and early tooth eruption among over-weight/obese children is clinically relevant for the dental profession, mainly for paediatric dentists and orthodontists.

In conclusion, this population-based study found a significant association between overweight/obesity at age 12 and early tooth eruption at ages 12 and 14-15 among school-children from southern Brazil. This finding was consistently found at both time points (baseline and follow-up) and irrespective of the outcome used to investigate such association (number of erupted permanent teeth, the presence of complete permanent dentition, the presence of erupted second molars at age 12, and the complete eruption of second mo-lars at age 14-15).
